# Supplementary material for: Coordinating multiple mental faculties during learning
Source: Sci Rep. 2025 Feb 13;15:5319. doi: 10.1038/s41598-025-89732-4 (PMC11822098; doi:10.1038/s41598-025-89732-4)
Supplement: Supplementary file 1 — Supplementary Information. [file 41598_2025_89732_MOESM1_ESM.pdf]

Coordinating multiple mental faculties during learning

Xiaoliang Luo[1*], Robert M. Mok[2,3], Brett D. Roads[1], Bradley C. Love[1,4]

[1]Department of Experimental Psychology, University College London, 26 Bedford Way, London, WC1H 0AP, UK
[2]MRC Cognition and Brain Sciences Unit, University of Cambridge, 15 Chaucer Rd, Cambridge, CB2 7EF, UK
[3]Department of Psychology, Royal Holloway, University of London Royal Holloway, University of London, Egham, TW20 0EX
[4]The Alan Turing Institute, 96 Euston Rd, London, NW1 2DB, UK

*To whom correspondence should be addressed; E-mail: xiao.luo.17@ucl.ac.uk

Supplementary Information

A Cluster solutions

Clusters \ Type	I	II	III	IV	V	VI
2	**71%**	–	–	3.8%	–	–
3	3.4%	–	–	–	–	–
4	18%	**67%**	7.0%	0.80%	1.6%	–
5	0.60%	3.4%	4.8%	6.0%	3.2%	–
6	1.8%	4.0%	**61%**	**65%**	**59%**	–
7	2.4%	6.6%	22.6%	22.8%	18.2%	3.0%
8	3.4%	19%	4.8%	1.8%	18%	**97%**

Table S.1: The clustering module of our model solves six category learning problems by recruiting varying number of clusters. The modal number of clusters recruited is 2, 4, 6, 6, 6, 8 for Type I–VI respectively, which is consistent with SUSTAIN.

B Alternative model results

We found that all three alternative models (Model 2-4; Fig. S.1) cannot account for human learning behavior of[1] and exhibit task-specific resource expenditure patterns found in[2]. While both Model 1 and 2 follow the costly-energy principle and show energy expenditure consistent with task difficulties, Model 2 exhibits unstable error trajectories towards the end of learning. A key reason for this pattern is because there is no pressure for the peripheral of Model 2 to respect representations of the controller, subsequently there is no constraint on how the DNN module learns, causing it to learn out-of-sync with the controller (leading to instability in how many clusters can the controller recruits; Table S.2). Both Model 3 and 4 show correctly ordered learning curves. However, neither of them exhibits consistent patterns of energy expenditure across problem types. In sum, both the controller-peripheral architecture and costly-energy principle are necessary components to facilitate coordination between the peripheral and controller module of the proposed model.

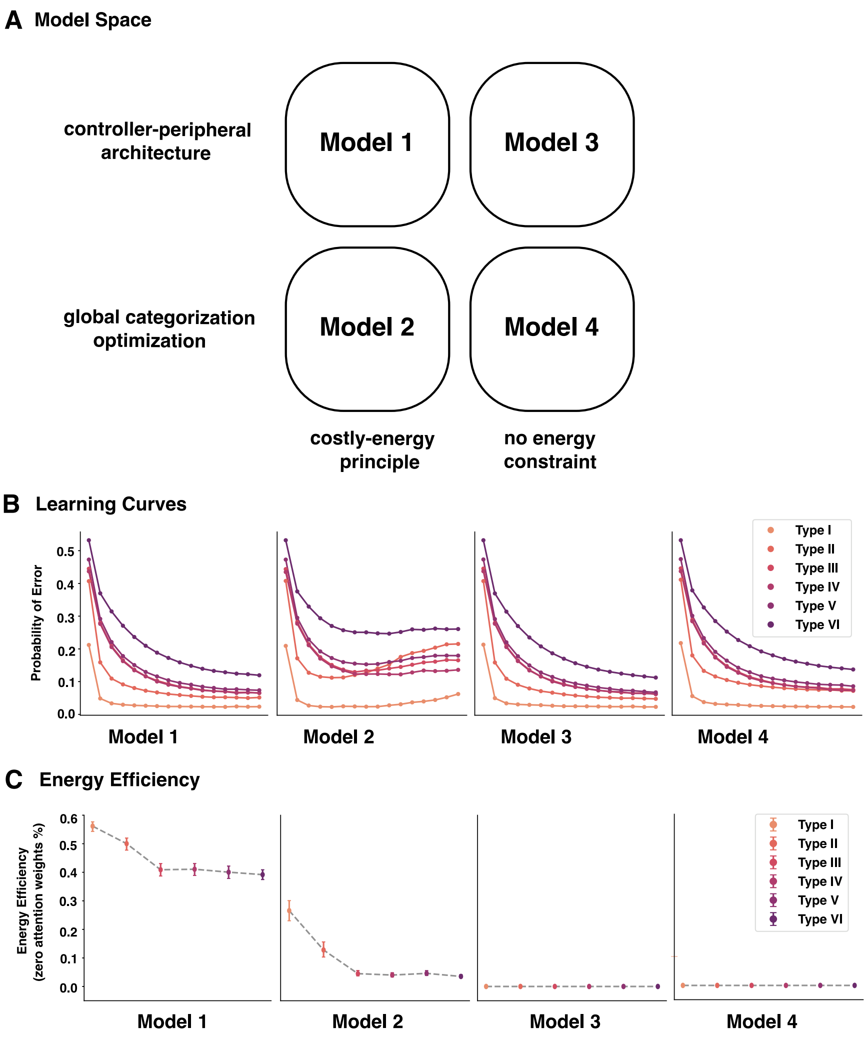
A Model Space
controller-peripheral architecture
global categorization optimization
Model 1
Model 3
Model 2
Model 4
costly-energy principle
no energy constraint
B Learning Curves
Probability of Error
0.0
0.1
0.2
0.3
0.4
0.5
Type I
Type II
Type III
Type IV
Type V
Type VI
Model 1
Model 2
Model 3
Model 4
C Energy Efficiency
Energy Efficiency (zero attention weights %)
0.0
0.1
0.2
0.3
0.4
0.5
0.6
Type I
Type II
Type III
Type IV
Type V
Type VI
Model 1
Model 2
Model 3
Model 4

Figure S.1: Comparison to alternative models in the model space. (A) Apart from our model (Model 1) which implements controller-peripheral framework and is optimized based on the costly-energy principle, we consider three alternative models which lack one or both elements. For Model 2 and 4 which lack the controller-peripheral architecture, they are optimized to minimize the standard global categorization error. For Model 3 and 4 which lack costly-energy principle, there are no constraints on the perceptual attention weights of their peripheral module; (B-C) All three alternative models (Model 2-4) cannot account for human learning behavior of[1] or show task-specific resource expenditure patterns as those found in[2]. For Model 2, while the energy expenditure (in terms of the sparsity of learned peripheral attention weights) across problem types is in the right order, it is not as significant as Model 1 and Model 2 cannot capture human behavior with global error optimization; For both Model 3 and 4, while they show correct difficulty ordering of six problem types, they do not demonstrate energy expenditure patterns that can reflect task difficulty.

Clusters \ Type	I	II	III	IV	V	VI
2	**59%**	–	–	2.6%	–	–
3	3.6%	–	–	–	–	–
4	19%	28%	3.0%	0.2%	–	–
5	1.0%	3.0%	2.6%	3.4%	1.4%	–
6	4.4%	9.0%	**47%**	**61%**	**48%**	–
7	3.4%	12%	34%	30%	24%	3.0%
8	8.8%	**47%**	13%	3.4%	27%	**97%**

Table S.2: Model 2 cluster recruitment.

Clusters \ Type	I	II	III	IV	V	VI
2	**71%**	–	–	4.2%	–	–
3	3.4%	–	–	–	–	–
4	18.2%	**69%**	7.4%	0.40%	1.6%	–
5	0.40%	3.2%	5.8%	6.6%	2.0%	–
6	2.2%	3.8%	**59%**	**65%**	**62%**	–
7	1.6%	7.0%	22%	22%	18%	3.6%
8	3.2%	17%	5.0%	1.6%	16%	**96%**

Table S.3: Model 3 cluster recruitment.

Type \ Clusters	I	II	III	IV	V	VI
2	**71%**	–	–	4.2%	–	–
3	2.4%	–	–	–	–	–
4	20.8%	**65%**	6.8%	0.20%	1.0%	–
5	0.40%	3.0%	5.2%	4.8%	1.8%	–
6	1.2%	4.4%	**61%**	**67%**	**62%**	–
7	1.6%	6.4%	22%	22%	19%	2.0%
8	2.8%	21%	4.8%	1.8%	17%	**98%**

Table S.4: Model 4 cluster recruitment.

C Attention compression two-way ANOVA results

	ddof1	ddof2	MS	F	P
Problem Complexity	2	42	41.27	78.18	< 0.001
Learning Block	15	315	0.66	28.07	< 0.001
Interaction	30	630	0.21	13.56	< 0.001

Table S.5: Two-way ANOVA showed significant main effects (Problem Complexity and Learning Block) as well as significant interaction between the two.

D Replicating using shape stimuli

Using image stimuli from[3] which have equivalent problem structures as the shape stimuli used in[4] as a replication of[1], our model obtain the same learning behaviors as shown in Fig. 2B. Here, we show when applied controller-peripheral architecture to the same shape stimuli, our model performs equivalently (Fig. S.2).

E Using Visual Transformer as peripheral

Visual transformers (ViT), a novel architecture relative to DCNNs, are actively studied for their neuroconnectionist ties[6]. Despite not being designed as a biologically-plausible model, ViT outperforms DCNNs in vision tasks, aligning more closely with human performance[7,8]. ViT-based architectures also excel in explaining neural responses in visual and other brain regions[9].

In our investigation, we explored integrating transformers into the controller-peripheral framework. Using a pretrained ViT as the peripheral, we faced initial challenges due to the absence of filters inherent in DCNNs. However, by draw-

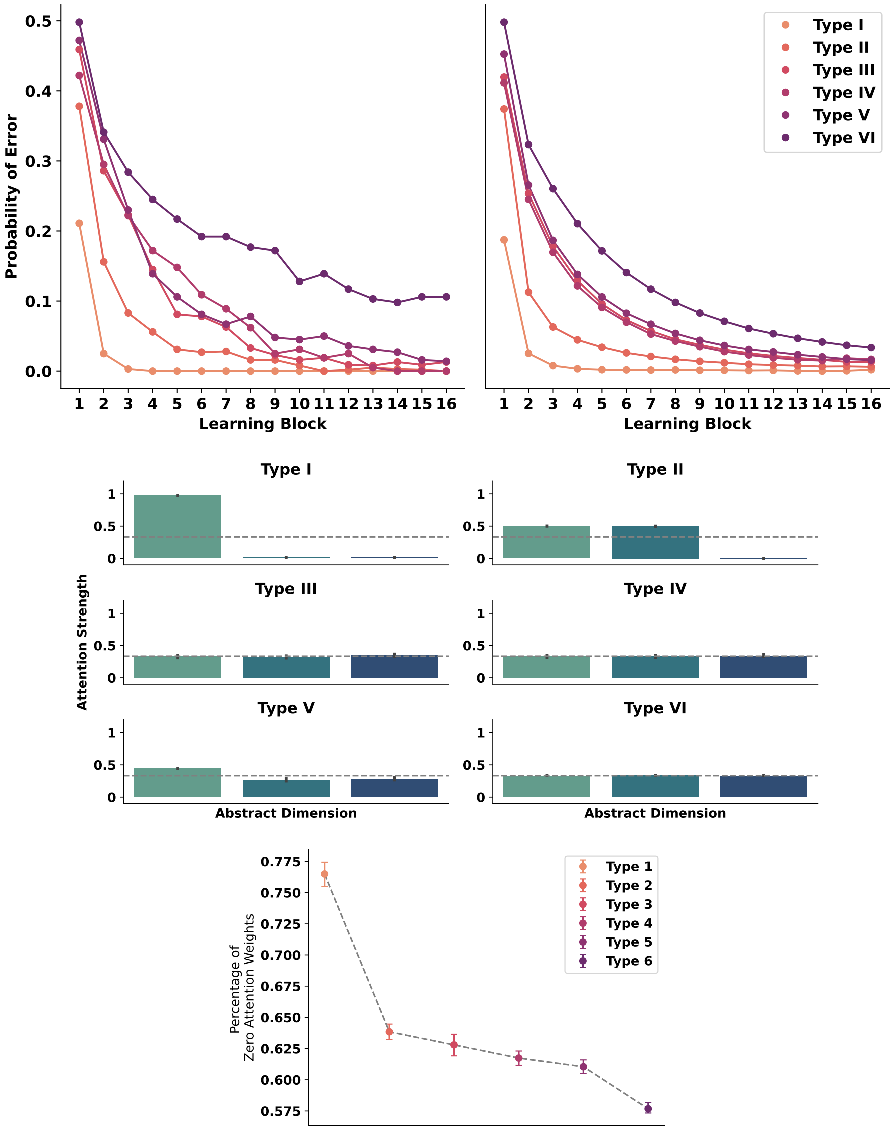

Figure S.2: (A) The model fits on the left were from[5]'s replication of[1]'s six categorization tasks using the simple shape stimuli as shown in Fig. 2A. (B) While we focus on the image stimuli from[3] of the same problem structure in our main experiments, we demonstrate here that the controller-peripheral framework obtain equivalent results using the shape stimuli.

ing an analogy between DCNN filters and transformer self-attention heads, we successfully applied attention modulation with an L_1 constraint.

We replicated key learning patterns of[4], suggesting the controller-peripheral framework's potential applicability across different model architectures. Future work could further explore which architectures are most effective, likely influenced by the specific data researchers aim to analyze. This understanding may enhance the framework's performance in diverse applications.

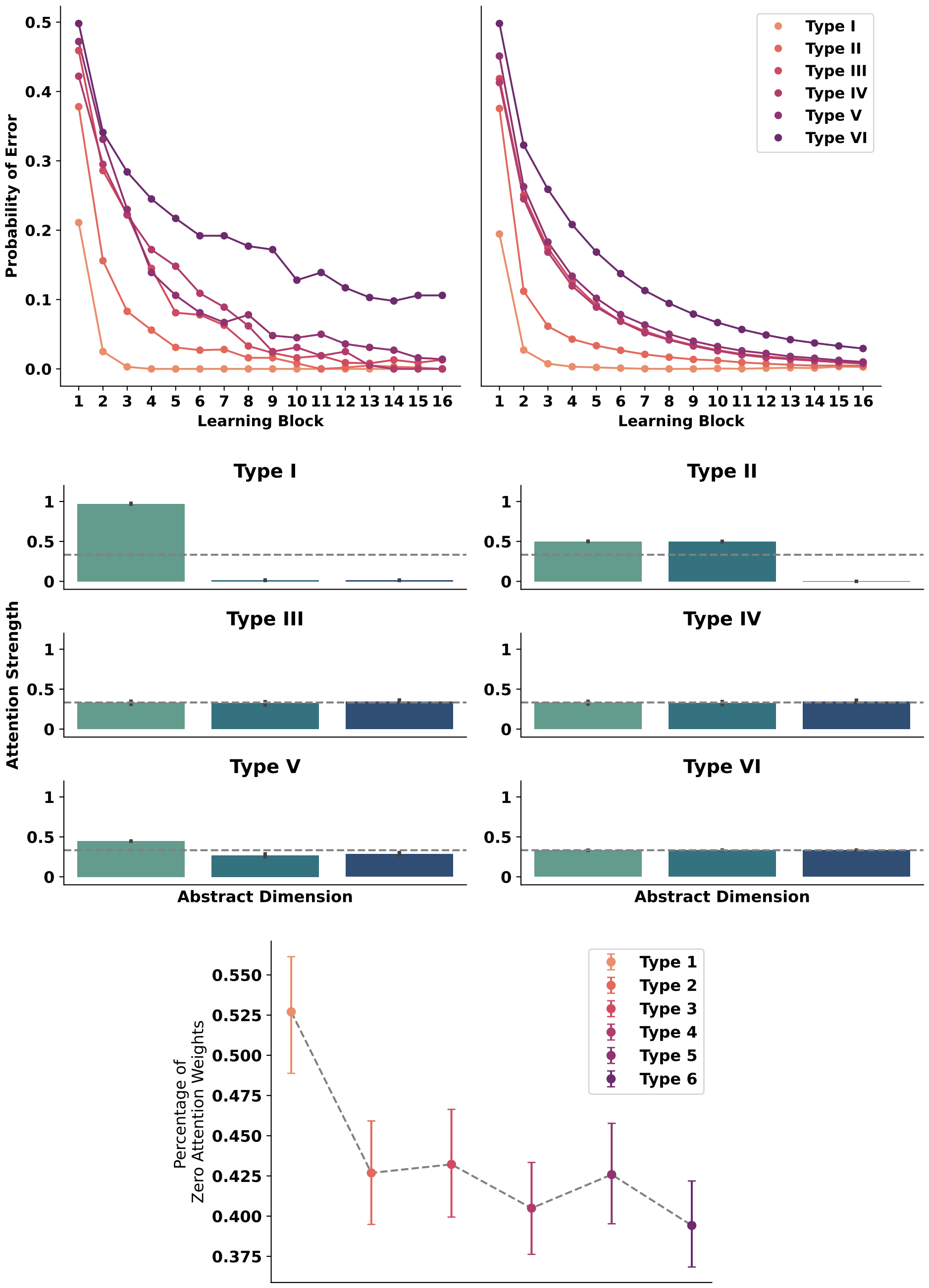

Figure S.3: Equivalent learning patterns are achieved using a visual transformer as the peripheral module of the controller-peripheral architecture.

F Fine-tuning results

Stimulus	Leg	Antenna	Mandible
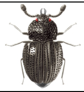	0	0	0
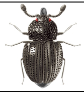	0	0	1
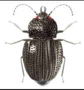	0	1	0
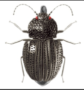	0	1	1
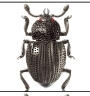	1	0	0
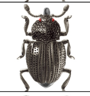	1	0	1
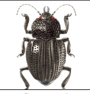	1	1	0
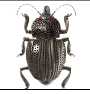	1	1	1

Table S.6: Mapping between pixel-level stimuli and binary-valued psychological representations.

Position \ Learning rate	$3e-3$	$3e-4$	$3e-5$	$3e-6$
fc2	0.833	1	1	0.833
block5_pool	1	1	1	1
block5_conv3	0.833	1	1	1
block5_conv2	1	1	1	1
block5_conv1	1	1	1	1
block4_pool	1	1	1	1
block3_pool	1	1	1	1

Table S.7: Performance from the first training procedure. Layer positions are listed (top to bottom) from advanced to intermediate.

Position \ Learning rate	$3e-3$	$3e-4$	$3e-5$	$3e-6$
fc2	0.643	0.661	0.679	0.625
block5_pool	0.839	0.768	0.768	0.750
block5_conv3	0.750	0.679	0.696	0.750
block5_conv2	0.893	0.857	0.893	0.946
block5_conv1	0.786	0.804	0.786	0.821
block4_pool	**0.964**	0.946	0.929	0.929
block3_pool	0.929	0.911	0.911	0.875

Table S.8: Performance from the second training procedure. Layer positions are listed (top to bottom) from advanced to intermediate.

G Controller-peripheral interaction overtime

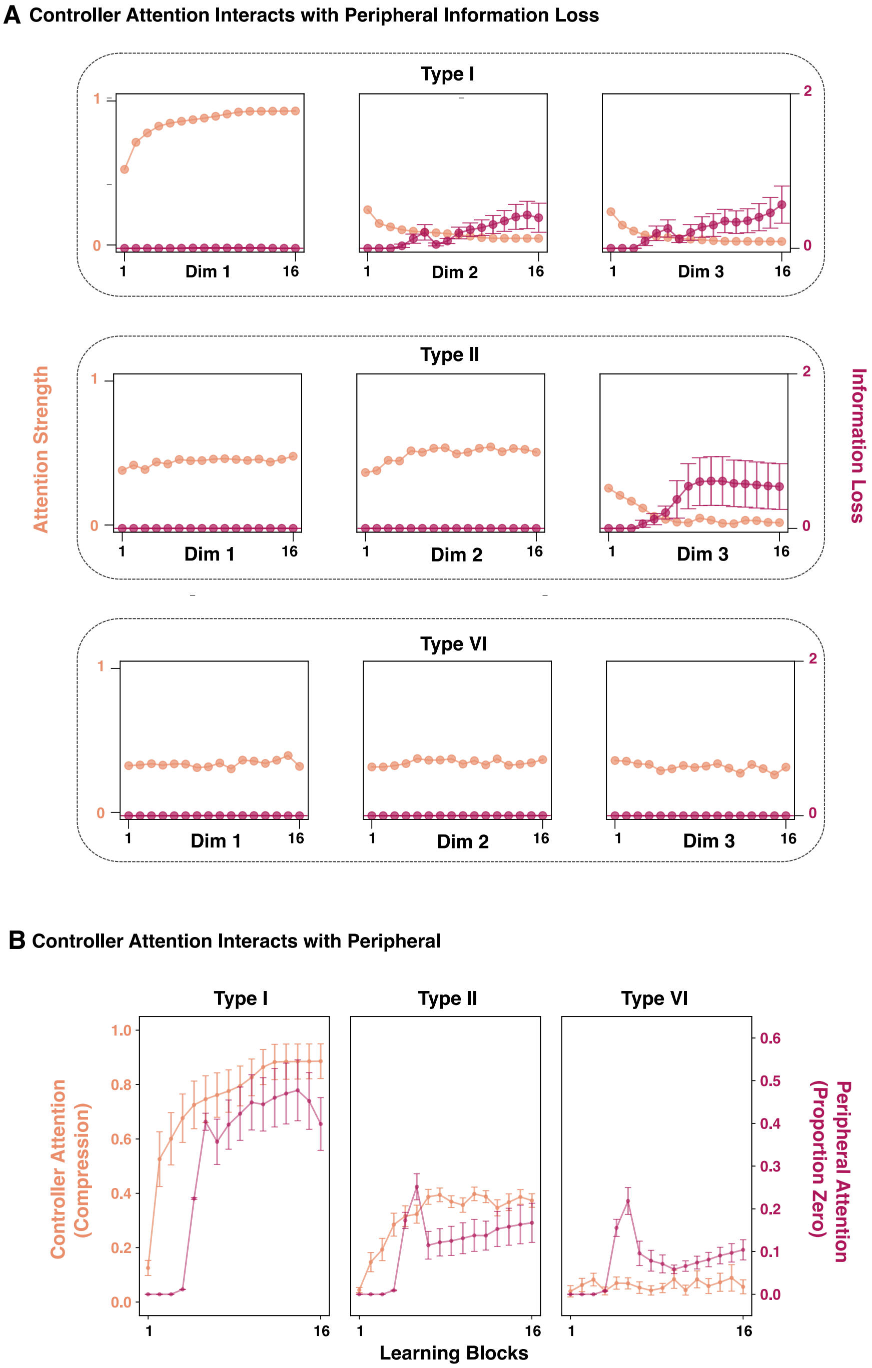

Figure S.4: Controller-peripheral interaction in the proposed model overtime. (A) Change in controller attention strength drives change in information loss of stimulus feature representations in the peripheral. For irrelevant dimensions of a problem type, decreasing controller attention strength leads to increasing information loss of the irrelevant dimensions; (B) The sparsity of peripheral attention weights corresponds to the compression level of controller attention weights. Decreasing compression level of controller attention over increasing task difficulty leads to decreasing sparsity level of peripheral attention weights.

H Participant performance varies when the relevant feature changes in Type I problem

We evaluated whether the three visual features of the stimulus (leg, antenna and mandible) were equally perceived by participants by measuring the difference in response time and categorization accuracy when different stimulus features are relevant for the task. We focused on Type I problem with only one relevant feature for the best contrast. We computed the average response time and accuracy over participants and learning blocks for each relevant feature. We found that when feature "mandible" was the relevant dimension, response time became significantly slower than when either of the other two dimensions was relevant. We also found that categorization accuracy was significantly lower when mandible was the relevant dimension than the other dimensions (Fig. S.5).

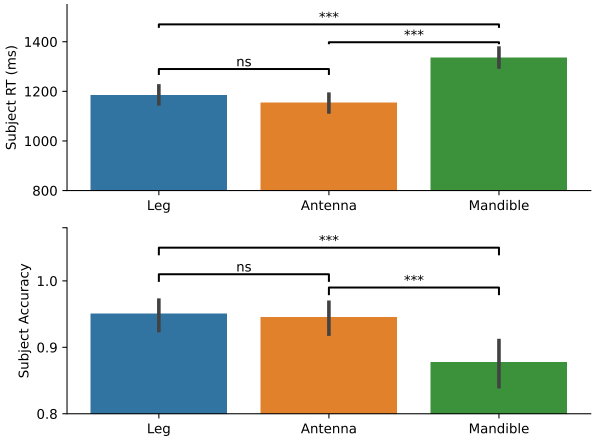

Figure S.5: Not all stimulus features were perceived equally by humans. We reviewed participant behaviors in Type I problem when only one feature is relevant and found that when "mandible" was the relevant feature, response time was significantly longer and categorization accuracy was significantly lower than when either of the other dimensions was relevant.

References

[1] Shepard, R. N., Hovland, C. I. & Jenkins, H. M. Learning and memorization of classifications. *Psychological Monographs: General and Applied* **75**, 1–42 (1961). URL `/record/2011-17802-001`.

[2] Ahlheim, C. & Love, B. C. Estimating the functional dimensionality of neural representations. *NeuroImage* **179**, 51–62 (2018).

[3] Mack, M. L., Love, B. C. & Preston, A. R. Dynamic updating of hippocampal object representations reflects new conceptual knowledge. *Proceedings of the National Academy of Sciences of the United States of America* **113**, 13203–13208 (2016).

[4] Nosofsky, R. M. Attention, similarity, and the identification-categorization relationship. *Journal of Experimental Psychology: General* **115**, 39–57 (1986).

[5] Nosofsky, R. M., Gluck, M. A., Palmeri, T. J., Mckinley, S. C. & Glauthier, P. Comparing modes of rule-based classification learning: A replication and extension of Shepard, Hovland, and Jenkins (1961). *Memory & Cognition* **22**, 352–369 (1994). URL `/record/1994-39915-001`.

[6] Doerig, A. *et al.* The neuroconnectionist research programme. *Nature Reviews Neuroscience* **24**, 431–450 (2023).

[7] Veerabadran, V. *et al.* Subtle adversarial image manipulations influence both human and machine perception. *Nature Communications* **14**, 1–12 (2023).

[8] Tuli, S., Dasgupta, I., Grant, E. & Griffiths, T. L. Are Convolutional Neural Networks or Transformers more like human vision? *Proceedings of the 43rd Annual Meeting of the Cognitive Science Society: Comparative Cognition: Animal Minds, CogSci 2021* **2**, 1844–1850 (2021).

[9] Berrios, W. & Deza, A. Joint rotational invariance and adversarial training of a dual-stream Transformer yields state of the art Brain-Score for Area V4. *arXiv* (2022). URL `http://arxiv.org/abs/2203.06649`.
